# Preclinical paired noninferiority study comparing in-house and commercially available 3D planning for corrective osteotomy of the distal radius

**DOI:** 10.1038/s41598-025-97788-5

**Published:** 2025-04-12

**Authors:** Charlotte Stor Swinkels, Katleen Libberecht, Emilia Gryska, Peter Axelsson, Per Fredrikson, Anders Björkman

**Affiliations:** 1 Department of Hand Surgery, Sahlgrenska University Hospital, Mölndal, Sweden; 2 Department of Medical Physics and Biomedical Engineering, Sahlgrenska University Hospital, Gothenburg, Sweden; 3 Institute of Clinical Sciences, Sahlgrenska Academy, University of Gothenburg, Gothenburg, Sweden

**Keywords:** 3D, Patient-specific, Osteotomy, Distal radius, In-house, Virtual planning, Bone, Biomedical engineering, Three-dimensional imaging, Orthopaedics

## Abstract

3D surgical planning and patient-specific guide design are becoming an established approach in complex skeletal surgery. Traditionally, this is outsourced to commercial companies, but an alternative is to establish an in-house hospital team for the process. This study aimed to compare the accuracy of in-house design with a commercial company. Sixteen patients with extra-articular distal radius malunions requiring surgery were included. A hospital-based team and a surgeon working with an external company independently planned surgery and designed guides for each patient. Accuracy was evaluated by comparing simulated corrections with the planned corrections using 3D-printed bone models. The null hypothesis was that the in-house guides were inferior to the externally purchased ones. Noninferiority margins of 5° for volar tilt and 2 mm for ulnar variance were set. The mean volar tilt error difference between the two guides was 2.3°, and the mean ulnar variance error difference was 0.38 mm, both within the noninferiority limits. The dimensional accuracy of the printed guides before and after sterilization showed minimal variation (less than 0.3 mm). The results demonstrated that in-house surgical planning and guide design for distal radius corrective osteotomies can achieve comparable accuracy to external commercial companies.

## Background

Fractures of the distal radius are the most common fractures in the body. In 2022, 17% of all fractures registered in the Swedish Fracture Register were wrist fractures^1^. Most distal radius fractures heal in its anatomically correct position; however, some result in malunion, leading to impaired function and pain^2,3^. Prior studies have shown that more accurate anatomical correction of distal radius malunions leads to better clinical outcomes^4,5^. Traditional techniques with two-dimensional planning of surgery on conventional radiographs and freehand repositioning often fail to achieve good results^4,6^. To improve the accuracy of the correction, the use of virtual planning on three-dimensional (3D) models and patient-specific surgical instruments (PSI), such as surgical guides, are becoming increasingly popular in complex skeletal surgery. Studies on the use of this concept in the correction of distal radius malunions report highly accurate results with low residual errors between the planned correction and the achieved surgical result^7–11^. Despite their availability for over 15 years and increased support in the literature, virtual 3D surgical planning and PSIs have yet to find their place in routine use in most hospitals.

Currently, there are two ways to use PSIs in clinical practice. The standard is to purchase the surgical plan and PSIs from external commercial companies. This approach is hindered by high costs and the need for external transfer of patient data. Additionally, successful 3D planning and guide design strongly relies on collaboration between surgeons and clinical engineers, which can be challenging to establish through web meetings with external companies. An alternative approach is a dedicated, in-house 3D planning unit that can help overcome several of these issues and make this technique more accessible. Moving the planning and design process into the hospital and creating a close, daily collaboration between surgeons and engineers has important advantages, but also places increased responsibilities on the staff and the need for a robust quality management system.

This study aims to assess whether in-house designed PSIs achieve noninferior^12,13^ results compared with externally purchased guides in a simulated extra-articular distal radius osteotomy model. An additional aim was to investigate the dimensional accuracy of the 3D-printed surgical guides and whether sterilisation had an impact on their form and shape.

## Materials and methods

### Study design

Sixteen consecutive adult patients who required a corrective osteotomy of an extra-articular malunion of the distal radius were prospectively enrolled at Sahlgrenska University Hospital (SUH) between 13th July 2021 and 13 July 2023. We selected osteotomy of extra-articular distal radius malunion as a model because the workflow for these corrections can be relatively standardised. The study was approved by the Swedish Ethics Review board (2021-01974) and performed in accordance with the European Union Medical Device Regulation 2017/745 and the Declaration of Helsinki. All patients gave written consent to participate after receiving oral and written information about the study. There were 14 women and two men with a median age of 61 years (range 21–77 years). Exclusion criteria were distal radius deformity in both arms, limited cognitive abilities, psychiatric disorders, active drug abuse and inability to read and understand Swedish. Patients with conditions that could impair bone healing were also excluded. Each patient underwent a bilateral high-resolution computed tomography (CT) scan of the entire forearm according to the scan protocol published by Materialise NV (Leuven, Belgium)^14^. The same CT scan was used for the standard technique and the in-house technique.

### 3D surgical planning by the external company

The patients’ CT DICOM images were sent to an external company, Materialise NV (Leuven, Belgium), specialised in 3D surgical planning. The engineers at the company segmented the images, proposed an osteotomy plan and designed the surgical guides according to the plan. The plan and guide design were discussed, adjusted and approved during at least two online meetings with an experienced hand surgeon (PA). Once the final planning and design were approved, 3D-printed surgical guides were shipped from the company to the hospital. Additionally, the plan was sent digitally to the hospital in the form of three STL-files containing the proximal radius, the distal repositioned radius and cylinders representing the location of the screws. The STL-files of the plate and the guides from the external company were not available for the study.

### In-house 3D surgical planning and guide design

**Fig. 1:**
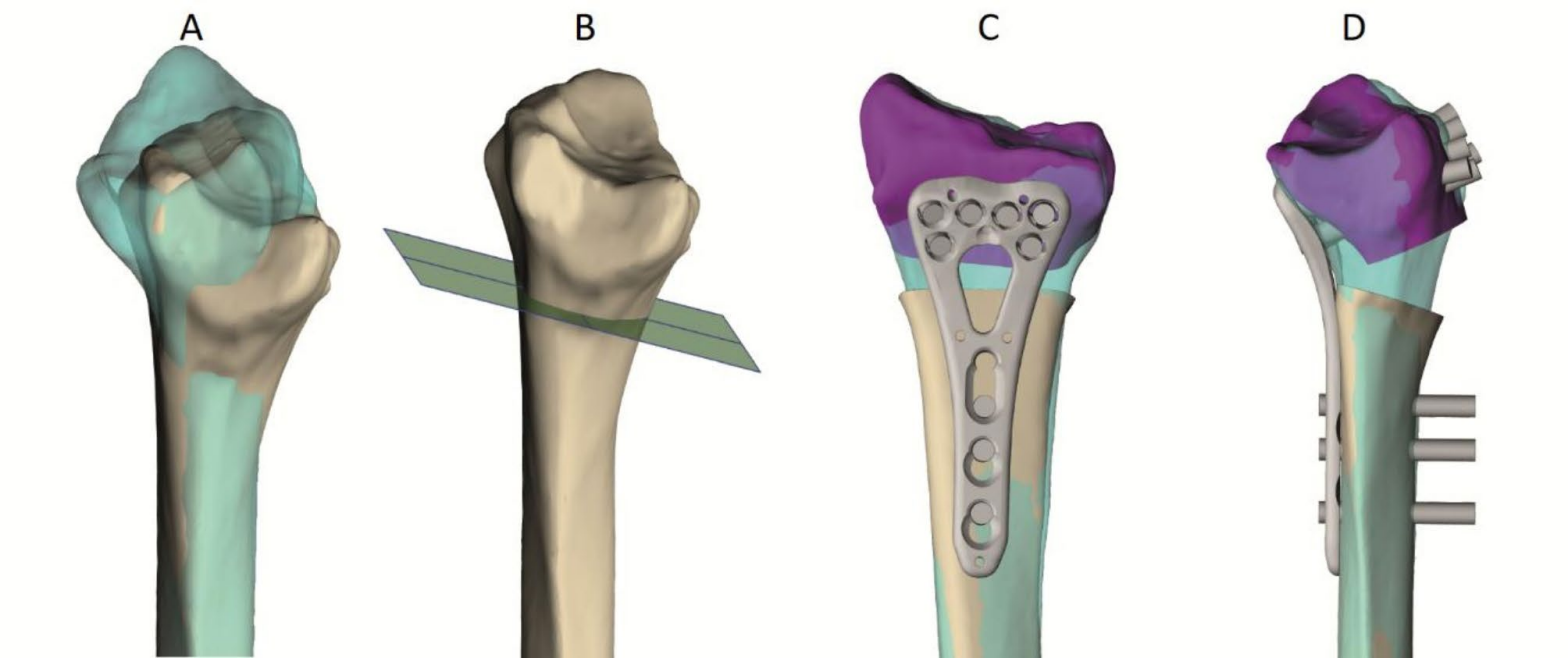
Virtual surgical planning. The blue model is the mirrored model of the healthy arm (**A**). The purple part of the radius is separated by the osteotomy plane and aligned with the healthy reference (**B**,** C**). The STL file of a plate is fitted to the corrected radius (**C**,** D**). (Materialise 3-matic Medical v17.0, https://www.materialise.com/en/healthcare/mimics/3-matic-medical).

The in-house SUH technique followed similar steps to the standard approach, however, the planning and guide design were done locally by a surgeon (KL) and hospital-based engineers (CSS and EG) using the commercially available Mimics Innovation Suite (MIS) software from Materialise NV (Leuven, Belgium) installed on hospital computers. In-house planning was conducted independently from the planning done by PA using Materialise, and often preceded the planning with the external company. The healthy and injured forearms were segmented by a hand surgeon or clinical engineer experienced in segmentation. 3D osteotomy planning was performed by a hand surgeon alone, or by an engineer in close collaboration with the surgeon (Fig. 1A–D).

**Fig. 2:**
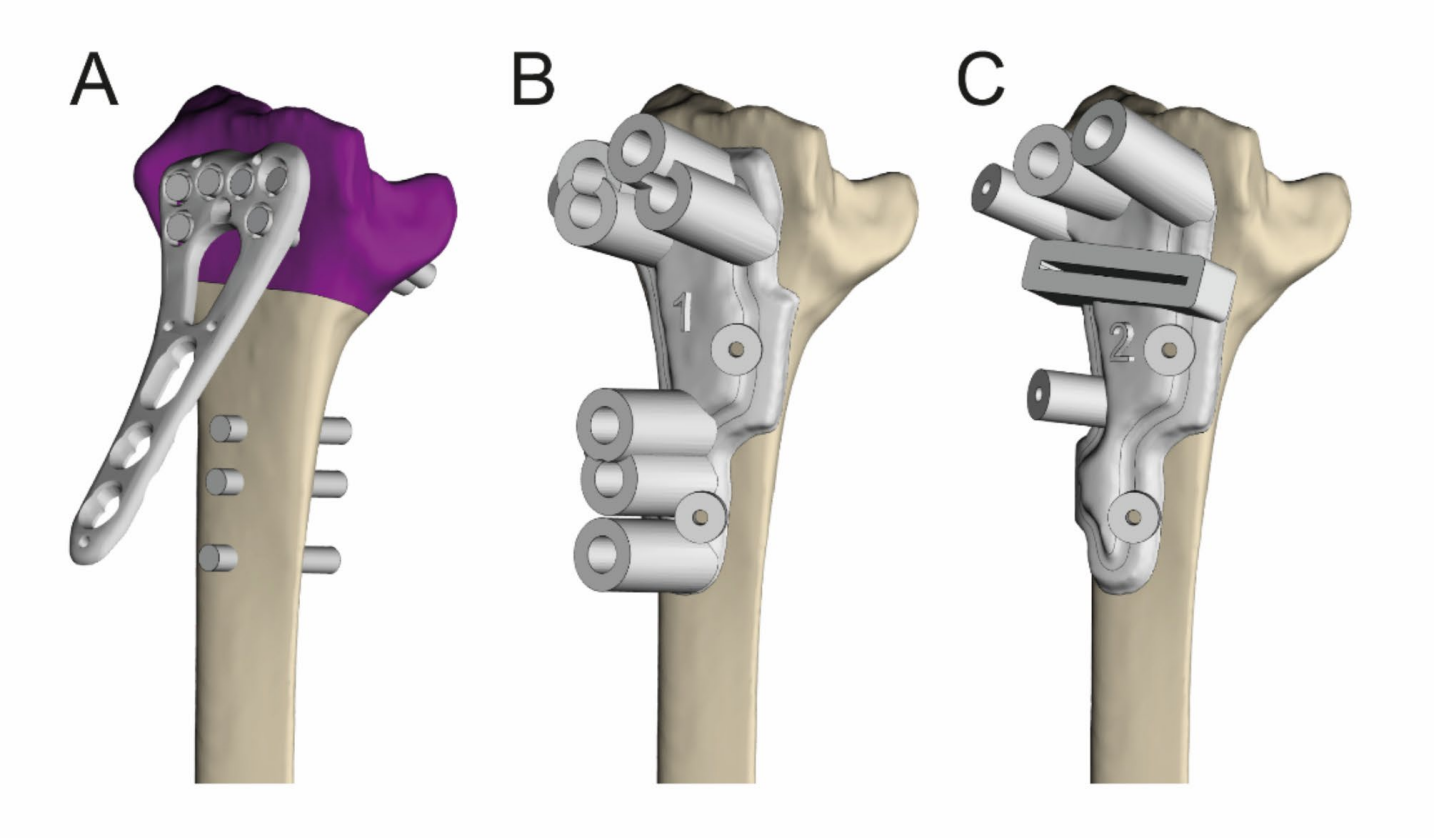
Guide design. The screws in the distal part of the radius were reversed along with the distal radius fragment to the original position of the radius (**A**). This determines the location of the drill holes, so that the final planned reduction is achieved when the plate is fixed with fixed-angle screws. Two surgical guides (**B**, **C**) were designed to guide the predrilling and cutting of the bone. (Materialise 3-matic Medical v17.0, https://www.materialise.com/en/healthcare/mimics/3-matic-medical).

The mirrored model of the healthy forearm was used as a reference for osteotomy planning. Digital models of osteotomy plates, which are essential for the virtual planning, were obtained from the manufacturer after signing of a non-disclosure agreement. The standard anatomical plates did not always fit well on the malunited bone. This was overcome with a combination of adaptation of the planning to the implant, plate bending and/or positioning of the plate with an offset distally, all done similarly by the in-house team and the external company. Design of a patient-specific set of surgical guides was done by a clinical engineer in collaboration with a surgeon based on the previously created surgery plan (Fig. 2A–C). The surgical guides consisted of features that determine the position and angulation of both the predrilled holes (drill cylinders) and the osteotomy (saw blade slot). Pre-drilling of screws was preferred instead of using k-wires as reference as the patient cases consisted mainly of dorsally angulated distal radius malunions in elderly patients with osteoporotic bones. The force required for reduction is substantial and the bone is weak, therefore it is safer to fixate all distal screws first to create a strong scaffold before levering the plate to the shaft of the radius.

After approval of the final design by the surgeon, the guides were optimised for 3D-printing, numbered and labelled. 3D-printing of the guides was done by an industrial partner according to the ISO13485 standard, while the hospital team remained the legal manufacturer. Two copies of the surgical guides were printed using a selective laser sintering (SLS) 3D printer (EOS P396), PA2200 MEDICAL raw material. The externally produced guides were manufactured in an equivalent manner.

### Surgery simulation and scanning

**Fig. 3:**
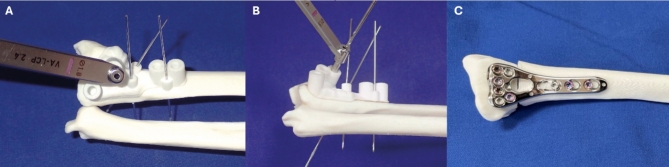
Surgery simulation. The drill cylinders were designed to fit the drill sleeve of the specific plate system used for the fixation (**A**, **B**). Surgery simulation result of a radius correction including plate (**C**).

Surgery was simulated on 3D-printed models of the malunited radius bone (Fig. 3). Two copies of each patient’s radius were printed in white polylactic acid (PLA) with a Fused Deposition Modelling 3D printer (Raise3D E2, Raise 3D Technologies Inc, Irvine, USA). During segmentation, a solid mask was created, so that only the outer surface of the radius constituted the 3D model. Slicing was done with ideaMaker 4.2.1 software (Raise3D E2, Raise 3D Technologies Inc, Irvine, USA). Print settings were 2.5 mm shell thickness, layer height of 0.15 mm and grid infill with a density of 25%, to assure adequate screw purchase in the plastic radius. An experienced hand surgeon (PA) simulated surgery for each patient on a 3D-printed model with externally designed guides and on a copy of the model with in-house designed guides. There was no defined order of the simulations. Both models were then scanned using an optical scanner (Einscan SP, Shining 3D® Tech. Co., Hangzhou, China) according to the instructions provided by the manufacturer.

### Transformation measurement

**Fig. 4:**
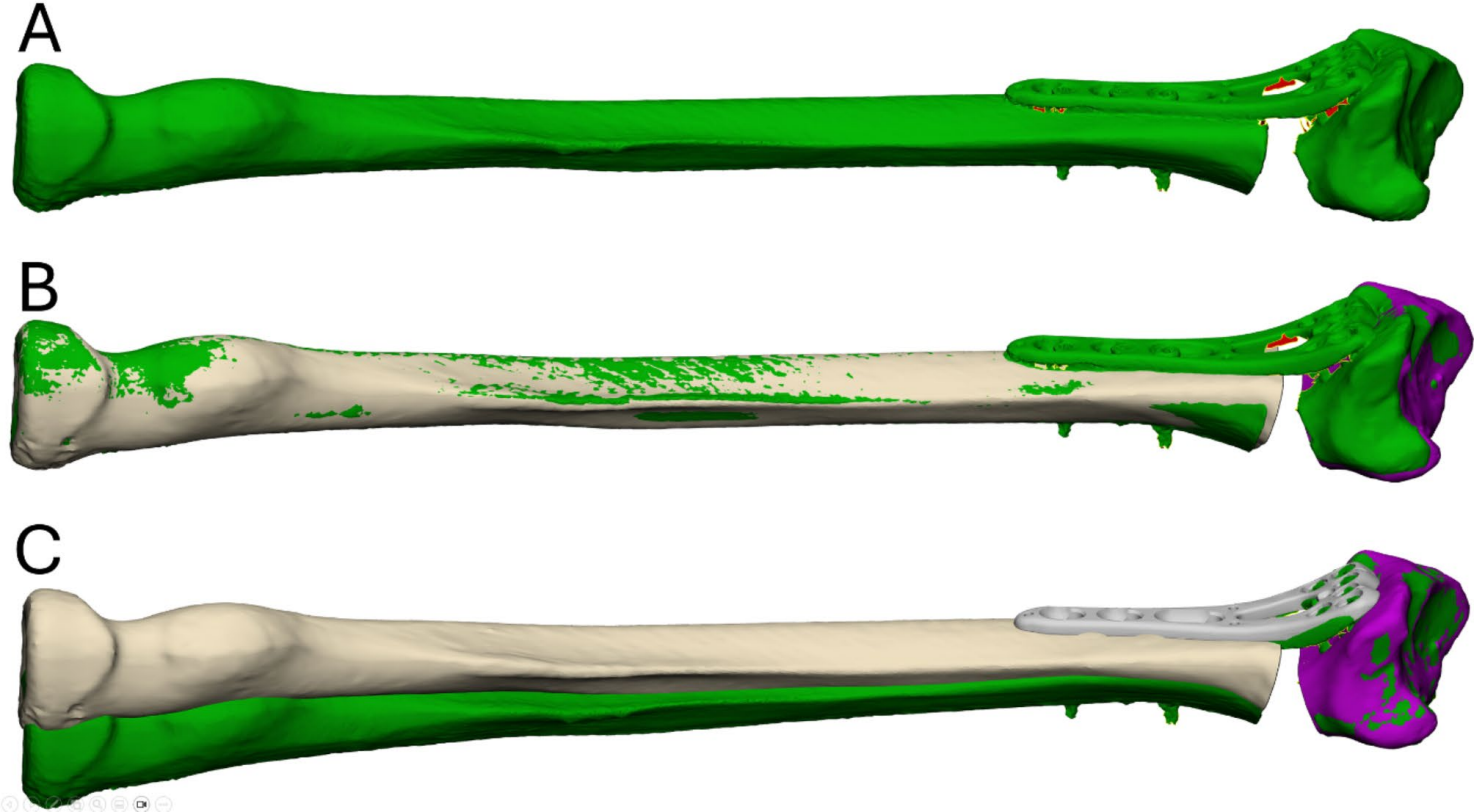
Transformation measurement. The scanned surgery result (green, **A**) was registered to the original plan (beige-purple) in the proximal radius (**B**). The transformation from plan to result was then measured in the distal radius (**C**). (Materialise 3-matic Medical v19.0, https://www.materialise.com/en/healthcare/mimics/3-matic-medical).

Using the MIS software, the proximal radius of the surgical result model was aligned to the proximal radius of the planned model (Fig. 4A, B) with the Iterative Closest Point algorithm (ICP)^15^. A 3D coordinate system was defined on the planned model and aligned with the world coordinate system, so that the origin became point (0,0,0). The distal part of the surgical simulation result model was then aligned to the distal radius of the planned model with the ICP algorithm (Fig. 4C). The transformation matrix in the world coordinate system presented the correction errors in six parameters according to the predefined 3D coordinate system.

### 3D coordinate system

**Fig. 5:**
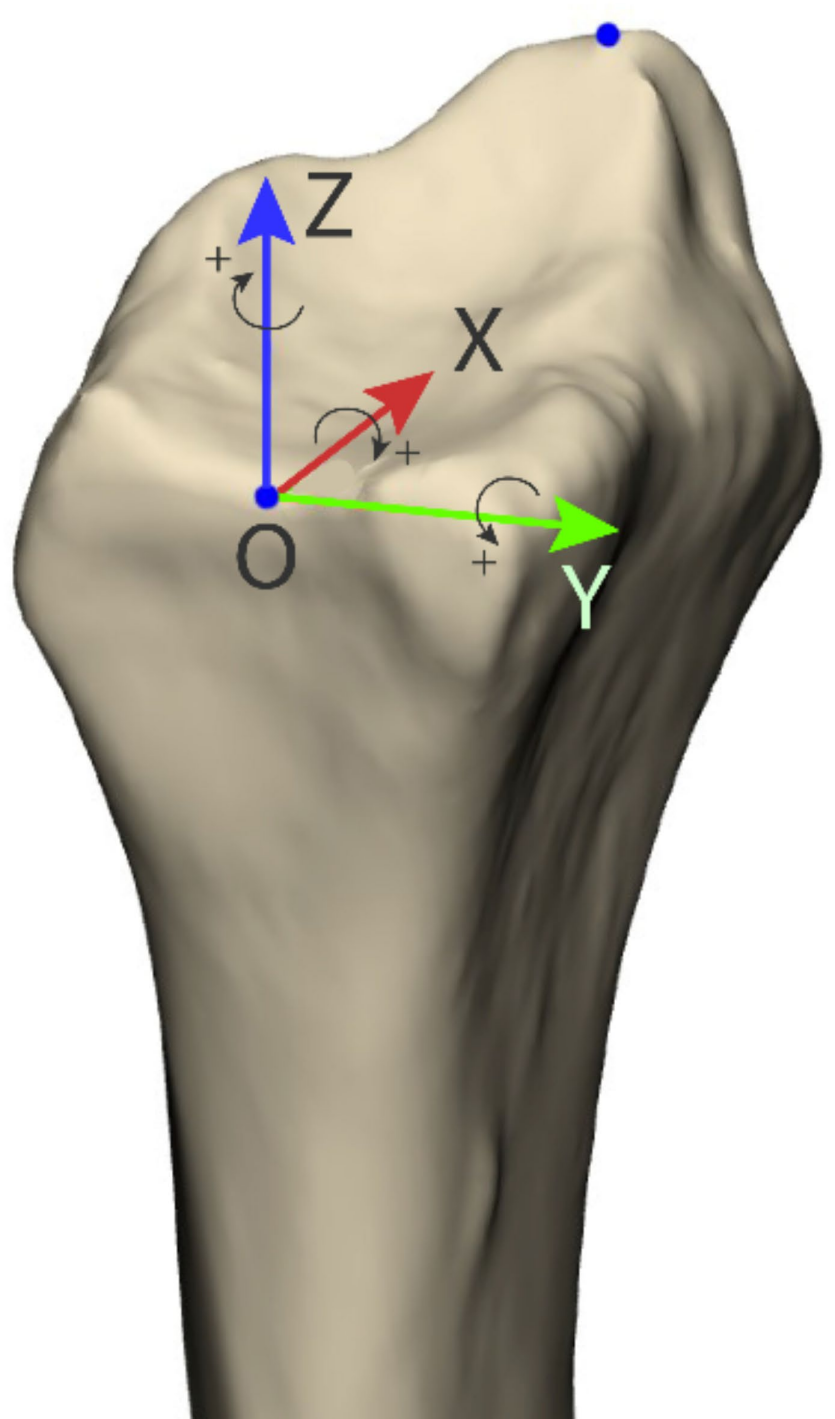
Anatomical coordinate system on a right-handed radius. The arrows on the axes indicate the direction for positive translation and the small arrows indicate the direction for positive rotation. The origin (O) is the middle of the rim between the sigmoid notch and the lunate fossa. The other blue dot indicates the tip of the styloid. (Materialise 3-matic Medical v17.0, https://www.materialise.com/en/healthcare/mimics/3-matic-medical, coordinate system visualisation added using Adobe Illustrator 27.6, https://www.adobe.com/products/illustrator.html).

The 3D coordinate system (Fig. 5) was established to align with recognized radiological parameters in three-dimensional space. Two anatomical key points were manually identified on the 3D model: the origin and the radial styloid. The origin was positioned at the centre of the rim between the lunate fossa and the sigmoid notch, resembling the Central Reference Point (CRP) used in conventional radiographs^16^. To define a central axis two planes were manually defined, perpendicular to the view in the anteroposterior and lateral position, on the segment between 3 and 5 cm proximal to the CRP^17,18^. The intersection of these two planes formed the central axis of the radius. A line from the origin, parallel to the central axis was defined as the z-axis. The x-axis was defined as a line perpendicular to the z-axis, from the origin to the projection of the tip of the radial styloid. The y-axis was the line from the origin perpendicular to the xz-plane.

The directions are shown in Fig. 5. In the measurement, the rotation in the x-axis represents the error in volar tilt, the rotation in the y-axis represents the error in radial inclination and the translation on the z-axis represents the error in radial length. The Y-axis is always positive in the volar direction, which means a mirrored anatomy will also mean a mirrored coordinate system. Rotation sequences follow the established methods used within radiology, to make them as clinically relevant as possible. The construction of the coordinate system and the measurements were all performed exclusively by an experienced surgeon (KL). Since the ulna remains unchanged in this model, the error in radial length measured at the rim of the sigmoid notch is equal to the error in ulnar variance. Lengthening of the radius is a positive value and volar rotation in the x-axis is a positive value.

### Statistical analysis

Since this was a noninferiority study, the null hypothesis (H0) stated that the new treatment (in-house SUH) was inferior to the standard treatment (external company, EC) by a pre-defined margin. The noninferiority margin was set to 5° volar tilt error and 2 mm radial length (ulnar variance) error. The margins were defined according to references of clinically relevant changes^2–4,6^. Since errors could have a negative or a positive value, and both are considered equally undesirable, absolute values were used.

$${\text{H}}0:\left| {{\text{error}}\;{\text{EC}}} \right| - \left| {{\text{error}}\;{\text{SUH}}} \right| \le - {\text{5}}^\circ \;{\text{and}}\; \le - {\text{2}}\;{\text{mm}}$$

The 95% confidence interval (CI) of the difference in volar tilt error and in radial length error were compared with the noninferiority margin. If the chance of the true mean error difference being within the noninferiority margin is more than 95%, H0 can be rejected and noninferiority can be claimed. In other words, if the lower limit of the 95% CI was within the noninferiority margins (i.e. higher than − 5° volar tilt and − 2 mm ulnar variance), the H0 was rejected. The transformation matrix provides translational and rotational errors in all three (XYZ) directions. However, for the noninferiority analysis, we focused on volar tilt and ulnar variance, as their clinically relevant error margins are well-defined, unlike the other errors.

### Dimensional accuracy of the printed guides

As potential geometrical errors in the manufacturing and/or sterilisation process could influence the results, we performed a validation on a subset of printed guides to rule out critical differences between the in-house and external manufacturing processes. The first five of the 16 cases were used as a sample to assess the print quality of the in-house designed surgical guides and the form stability after sterilisation for both in-house and external guides. These five cases included 13 in-house designed guides and 18 externally designed guides, a total of 31 guides (number of guides per patient varied). The printed guides were scanned using the same optical scanner before and after sterilisation. The surgical guides were sterilised using an autoclave machine according to the hospital protocol, 4 min at 134 °C and 1.5 bar, followed by a drying period. The optically scanned pre- and post-sterilisation guide models were aligned to the designed virtual model in the MIS software with the ICP algorithm. The average distance error (ADE) is returned by the software which represents the fitting accuracy of the two surfaces. For the externally designed guides, only the pre- and post-sterilisation printed guides were compared since the original design files were not available. Optical scanning can only scan the outer visible surfaces. Therefore, the calculation of the ADE was only done on the outer surfaces, consisting mainly of the contact area with the bone and the outer surface of the drill cylinders. These surfaces were marked on the scanned guides and registered as moving entities to the original file, defined as fixed entities, with the automatic distance threshold. The differences between pre- and post-sterilisation results of in-house guides and external guides were measured. A Shapiro-Wilk test was performed on the data to assess normality. As the data was not normally distributed the significance between the two data sets was analysed using a non-parametric Mann-Whitney U-test (SPSS Statistics, IBM, version 30.0). Finally, a post-hoc power test was performed using G*Power (Universität Kiel, Germany, version 3.1.9.7).

## Results

### Noninferiority analysis

The resulting transformations according to the coordinate system are summarised in Table 1. The mean difference between the rotation errors in volar tilt (x-axis) of the in-house guides and the externally designed guides was 2.3° (95% CI 0.6°–4.0°). The mean difference between the translation errors in ulnar variance (z-axis) of the in-house guides and the external guides was 0.38 mm (95% CI 0.11–0.66 mm).

**Table 1:** Rotational and translational errors between the resulting correction and the planned correction in absolute values. Rotation in X corresponds to the error in volar tilt correction. Translation in Z corresponds to the error in ulnar variance correction. Rotation in y corresponds to the error in radial inclination correction.

	Rotation error, mean (SD), °		Translation error, mean (SD), mm	
	In-house	External	In-house	External
x	2.61 (1.39)	4.93 (3.47)	0.9 (0.74)	1.28 (0.89)
y	1.96 (1.19)	3.13 (1.61)	0.98 (0.86)	1.08 (0.89)
z	3.39 (2.81)	3.96 (3.28)	0.9 (0.59)	1.29 (0.80)

**Fig. 6:**
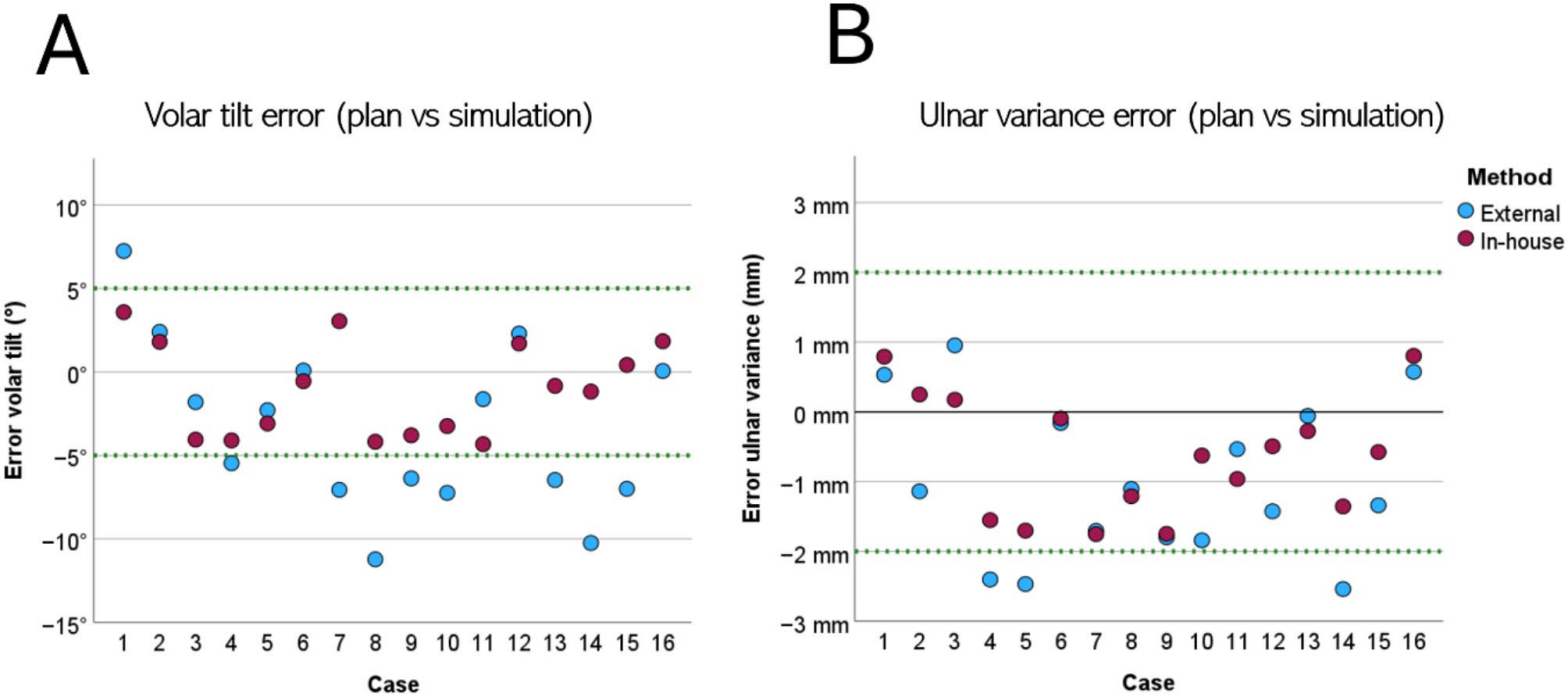
Scatter plot displaying in-house versus external deviation of virtual plan versus simulation results in volar tilt (**A**) and ulnar variance (**B**). The non-inferiority margins of 5° volar tilt and 2 mm ulnar variance being the limits for acceptable results (green dashed line).

The complete CIs lie within the noninferiority margins for volar tilt and for ulnar variance. Thus, the null hypothesis for both measures can be rejected and noninferiority can be claimed for the in-house guides. A scatter plot displaying the deviation between surgery simulation and virtual planning in volar tilt and ulnar variance for each patient case, including non-inferiority margins, is shown in Fig. 6.

### Analysis of dimensional accuracy of the surgical guides

The surface comparison of the printed in-house guides with the design files revealed an ADE of 0.27 ± 0.085 mm. The comparison before and after sterilisation revealed an ADE of 0.085 ± 0.007 mm for the in-house guides and 0.091 ± 0.013 mm for the external guides. The Mann-Whitney U-test (*p* > 0.05) showed that there was no significant difference in ADE between the two types of guides (*p* = 0.115). The post-hoc power for this test was 0.35 (*N* = 31), which means that there is a 35% chance to discover a difference between the groups.

The average distance errors and standard deviation between the virtual guide models and printed models scanned before and after sterilisation are shown in Table 2.

**Table 2:** Dimensional accuracy of printed guides for 5 cases before and after sterilisation.

	Mean average distance errors (SD), mm	
	In-house (*n* = 13)	External (*n* = 18)
Virtual versus pre-sterilisation model	0.27 (0.085)	N/A
Pre- versus post-sterilisation model	0.085 (0.007)	0.091 (0.013)

## Discussion

This study showed in preclinical testing that a dedicated in-house team can virtually plan and design patient-specific surgical guides for corrective osteotomies of the distal radius with noninferior results compared with commercially acquired.

The coordinate system that we used aims to measure rotations and translations in 3D space that resemble the standard measurements on conventional radiographs, like volar tilt, radial inclination and ulnar variance. These measures are all based on the CRP^16^ on conventional radiographs. By placing the origin of the coordinate system on the ridge of the sigmoid notch, we can define clinically relevant acceptable limits as noninferiority margins^2–4,6^.

When comparing our results with prior studies, across publications, there are differences in methodology, measuring tools and definition of the 3D coordinate systems^7,9,11,19^. The most similar measurement is the volar tilt or flexion-extension error, allowing for comparison across studies. In a clinical study, Vlachopoulos et al. reported a residual error in the flexion-extension rotation of 3.84° in seven patients who underwent 3D planned and guided extra-articular distal radius osteotomy^11^. The same research group later published the results of a technically improved guide design and found a residual flexion-extension mean rotational error of 1.97°, when calculated on absolute values^7^. These results compare well to our results, however, the results of Roner et al.^7^ were from a clinical study, obviously adding to the complexity.

Assessment of the print quality of the surgical guides showed that the manufacturing process of the in-house designed guides had an ADE of 0.27 mm when comparing an optical scan of the 3D-printed guides with the original CAD files. This error partly reflects the manufacturing process, but also the scanning resolution. Printing was done with a 0.12 mm layer thickness. Typically, SLS printing has a resolution of 0.1 to 0.2 mm^20^. The Einscan SP scanner has a single-shot error of ≤0.05 mm and a point distance of 0.17–2 mm according to the manufacturer’s technical specifications. The main disadvantage of optical scanning is that only the outer surface of the object can be scanned. Since the surgical guides are very complex structures with long cylinders, optical scanning is suboptimal. The advantages of optical scanning are the high resolution and the safety and accessibility in a clinical setting. We find it sufficiently accurate for quality control of 3D prints in our clinical practice and did not expect any difference between in ADE the in-house and the externally produced guides before and after sterilisation, which was confirmed by the Mann-Whitney U-test. A study comparing 3D-printed mandibles with the 3D model file by using the same scanner as the current study found a Root Mean Square (RMS) error of 0.11 mm for SLS printing on an EOSINT P385 printer^21^.

Some of the advantages of an in-house approach are that there is no need for external transfer of patient data. The technology is more accessible as the technical competence is physically in the hospital. Additionally, the skills of virtually planning surgeries and designing surgical guides stay within the hospital. In this study, costs for in-house planning and surgical guides were substantially lower compared to an external commercial company. We have estimated the cost of each in-house designed patient case to 700 EUR/case including work hours for both engineers and surgeons, hardware leasing and software licences. In addition, costs for manufacturing of surgical guides by an ISO13485 certified industrial 3D printing partner was approx. 400 EUR/case. Adding up to a total of 1,100 EUR/case using the in-house technique. At the time of the study cost per case by external commercial company was in the range from 2600 EUR/case to 3,800 EUR/case. A comprehensive cost-analysis including costs for infrastructure start-up, training of engineers and surgeons etc. was not covered by this study. However, a literature-based financial analysis was performed by Ballard et al., in which cost-savings were estimated based on surgery time versus fixed costs of in-house printing facilities. The study concluded that approximately 63 anatomical models or surgical guides per year is the minimum for a hospital to cover the annual fixed costs of an in-house printing facility^22^.

This study has several limitations. The construction of the 3D coordinate system relied on operator input as the method involved manual selections of anatomical key points. While the process was consistently performed by the same experienced operator, ensuring systematic application, the potential for human error remains, and its extent has not been quantified. Future studies could explore more automated and/or mathematically defined approaches to improve repeatability and reduce operator dependence.

Additionally, the surgery simulations were done on radius models, segmented by the in-house team. These are essentially the same models that were used for the planning and design of the in-house guides. This could introduce a bias since the externally designed guides were designed on another segmentation of the same radius and might therefore not fit as well to the in-house segmented radius. However, all segmentations aim to represent the true radius, but the differences in baseline models should be documented in future studies. Another limitation was that this study was done on extra-articular distal radius malunions, which are relatively easy to correct. The process is fairly standardised, and one could argue that similar results would be reached regardless of the technique. Additional studies are needed to assess if similar results can be achieved for more complex skeletal reconstructions. A future study will evaluate the clinical outcomes of the same patients one year after surgery using in-house designed surgical guides.

## Conclusions

In this study we showed that in-house 3D surgical planning and guide design can be a viable and accurate alternative to outsourcing to external industrial partners. In addition, this study provides evidence that our method of simulated surgery on 3D-printed anatomical models can be used to ensure the quality of the in-house design process without the need for comparison with commercially produced guides.

## Data Availability

The datasets supporting the conclusions of this article are available from the corresponding author on reasonable request.
